# An Expendable Player in Positive Vascular Remodeling? ADAMTS13 Deficiency Does Not Affect Arteriogenesis or Angiogenesis

**DOI:** 10.3390/ijms26189137

**Published:** 2025-09-19

**Authors:** Carolin Baur, Amanda Geml, Kira-Sofie Wimmer, Franziska Heim, Anja Holschbach, Katharina Elbs, Michael R. Rohrmoser, Dominic van den Heuvel, Alexander T. Bauer, Stefan W. Schneider, Daphne Merkus, Elisabeth Deindl

**Affiliations:** 1 Institute of Surgical Research, Walter Brendel Centre of Experimental Medicine, University Hospital, Ludwig-Maximilians-Universität München, 81377 Munich, Germany; carolin.baur@med.uni-muenchen.de (C.B.); amanda.geml@med.uni-muenchen.de (A.G.); kira.wimmer@med.uni-muenchen.de (K.-S.W.); franziska.heim@med.uni-muenchen.de (F.H.); katharina.elbs@med.uni-muenchen.de (K.E.); michael.rohrmoser@med.uni-muenchen.de (M.R.R.); dominic.van@med.uni-muenchen.de (D.v.d.H.); daphne.merkus@med.uni-muenchen.de (D.M.); 2 Biomedical Center, Institute of Cardiovascular Physiology and Pathophysiology, Faculty of Medicine, Ludwig-Maximilians-Universität München, 82152 Planegg-Martinsried, Germany; 3 Department of Medicine I, Ludwig-Maximilians-University School of Medicine, 81377 Munich, Germany; 4 Department of Dermatology and Venereology, University Medical Center Hamburg-Eppendorf, 20246 Hamburg, Germany; a.bauer@uke.de (A.T.B.); st.schneider@uke.de (S.W.S.); 5 Division of Experimental Cardiology, Department of Cardiology, Thoraxcenter, Erasmus MC, University Medical Center Rotterdam, 3015 GD Rotterdam, The Netherlands

**Keywords:** arteriogenesis, angiogenesis, ADAMTS13 deficiency, thromboinflammation, von Willebrand factor (VWF)

## Abstract

Peripheral artery disease is a common manifestation of atherosclerosis, characterized by insufficient tissue perfusion and chronic ischemia. Arteriogenesis and angiogenesis are essential endogenous mechanisms to restore blood flow and limit ischemic injury. The metalloprotease ADAMTS13, known for cleaving ultra-large von Willebrand factor, has been implicated in thrombotic and inflammatory regulation. However, its role in ischemic vascular remodeling remains unclear. Using a murine hind limb ischemia model, we investigated the effect of ADAMTS13 deficiency on arteriogenesis and angiogenesis by comparing male ADAMTS13^−/−^ and wild-type control mice. Perfusion recovery, vascular cell proliferation, immune cell infiltration, and thrombotic activity were evaluated using laser Doppler measurements, immunohistochemical analysis of adductor and gastrocnemius muscle tissues, and in vivo microscopy. ADAMTS13 deficiency did not impair perfusion recovery, collateral artery growth, or capillarization. While platelet adhesion was slightly increased in ADAMTS13^−/−^ mice, no thrombotic occlusions were observed. Inflammatory responses, including macrophage and neutrophil infiltration as well as macrophage polarization, were largely unaffected. Despite previous in vitro evidence indicating an angiogenic role for ADAMTS13, its absence did not compromise angiogenesis in vivo. Our findings suggest that ADAMTS13 does not play a critical role in ischemia-related angiogenesis and arteriogenesis under sterile conditions and may be relevant only in contexts involving acute and sufficiently strong thromboinflammatory stimuli.

## 1. Introduction

Atherosclerosis is a major global contributor to morbidity and mortality, affecting both coronary and peripheral arteries. It contributes to a range of ischemia-related diseases, including coronary artery disease, stroke, and peripheral artery disease (PAD), all resulting from plaque buildup and arterial narrowing. Despite surgical treatment options like bypass surgery and recent advances in percutaneous interventions to restore blood flow, PAD remains a major global health issue, especially in aging populations, with only limited options for noninvasive therapies. PAD affects more than 200 million people globally and is primarily caused by arterial obstruction, leading to ischemia and tissue necrosis, particularly in the lower limbs [1]. The body, however, attempts to compensate for the loss of an arterial vessel by developing collateral arteries that serve as a natural bypass. This process, known as arteriogenesis, which refers to the growth of pre-existing collateral vessels into functional arteries, is characterized by increases in lumen diameter and vessel wall thickness [2].

Severe obstruction of a femoral artery results in redirection of blood flow into pre-existing arterioles that connect the proximal and distal branches of the blocked artery. This increases fluid shear stress, the frictional force of blood flow against the vessel wall sensed by endothelial cells, which is the primary stimulus for arteriogenesis [2]. It initiates a signaling cascade leading to endothelial activation, leukocyte extravasation, and vascular cell proliferation, ultimately causing vascular remodeling and restoration of perfusion [3]. Shear stress also induces the release of extracellular RNA from endothelial cells, which enhances the binding of vascular endothelial growth factor (VEGF) to the co-receptor neuropilin-1, thereby promoting vascular endothelial growth factor receptor 2 (VEGFR2) signaling [4,5]. This activation promotes exocytosis of Weibel-Palade bodies and the release of von Willebrand factor (VWF) during the process of arteriogenesis [4]. While parts of VWF are released into the circulation, some molecules remain anchored to endothelial cells [6]. Due to high shear stress, platelets can transiently bind to VWF via their glycoprotein Ibα (GPIbα) receptor, leading to platelet activation and the formation of platelet-neutrophil aggregates (PNAs), a key step in the inflammatory response during arteriogenesis [7,8]. PNA formation is followed by leukocyte extravasation [8]. Neutrophils generate reactive oxygen species, which activate mast cells and increase the bioavailability of cytokines, thereby leading to the recruitment of monocytes [9,10]. These monocytes differentiate in the perivascular space into macrophages that release matrix metalloproteinases, cytokines, and growth factors, collectively stimulating vascular growth and contributing to the expansion of the collateral vessel wall and lumen [11,12,13,14]. Both pro-inflammatory M1-like and pro-regenerative M2-like macrophages are involved, with M2-like macrophages predominating in the later stages to promote vascular remodeling [15,16].

Unlike arteriogenesis, angiogenesis refers to the formation of new capillaries through sprouting or intussusception from pre-existing vessels [2,17]. It is primarily driven by hypoxia, which transcriptionally upregulates VEGF expression, a potent angiogenic factor [17,18]. VEGF-A promotes endothelial cell differentiation, proliferation, and remodeling through its interaction with VEGFR-2 [18]. Leukocytes, particularly neutrophils and monocytes, also play a critical role by supplying VEGF [19]. In addition to growth factor production, macrophages and neutrophils contribute to angiogenesis by supporting endothelial cell activation, matrix remodeling, and stabilization of vessel connections [20,21]. Thus, leukocytes are essential not only for clearing cellular debris at ischemic sites but also for remodeling the surrounding matrix and regulating the balance of pro- and anti-angiogenic factors [18,21].

ADAMTS13 (A Disintegrin and Metalloprotease with Thrombospondin type 1 repeats, member 13) is a zinc-dependent metalloprotease that cleaves VWF [22]. It is mainly produced by hepatic stellate cells, but also by endothelial and smooth muscle cells [23,24,25].

VWF is secreted as ultra-large multimers (ULVWF) from Weibel–Palade bodies and platelet α-granules [26,27,28,29]. Under conditions of high shear stress, such as in arterioles or collateral vessels, ULVWF unfolds, exposing platelet-binding sites and the cleaving site for ADAMTS13 [30,31]. By processing ULVWF into smaller, less thrombogenic forms, ADAMTS13 regulates the multimer size of VWF and thereby reduces its pro-thrombotic potential [6,31,32,33]. Consistent with this role, ADAMTS13 deficiency causes thrombotic thrombocytopenic purpura (TTP) in humans, a disorder characterized by widespread microvascular thrombosis due to uncleaved ULVWF [22,34]. Emerging evidence shows that ADAMTS13 has broader roles in vascular homeostasis, including antithrombotic and anti-inflammatory effects. In several disease models, ADAMTS13 deficiency has been linked to increased leukocyte recruitment and infarct size, while recombinant ADAMTS13 treatment was shown to attenuate the expression of adhesion molecules and pro-inflammatory chemokines, suggesting a regulatory role in sterile inflammation [35,36,37,38,39,40,41,42].

Despite the molecular evidence supporting a role for ADAMTS13 in both hemostatic and inflammatory pathways, its specific contribution to arteriogenesis and angiogenesis has not been systematically studied in vivo. Given the inflammatory nature of both processes, we hypothesized that ADAMTS13 may modulate vascular adaptation following arterial occlusion. During arteriogenesis, the release of highly reactive ULVWF from endothelial cells, combined with platelet activation under inflammatory conditions, creates a potentially pro-thrombotic environment in which thrombus formation could theoretically occur. However, despite extensive study, thrombotic occlusion of collateral vessels has not been observed. It has been proposed that ADAMTS13 mitigates this thrombotic risk by cleaving ULVWF under high shear conditions in the presence of platelets [33].

To address a potential role for ADAMTS13 in arteriogenesis and angiogenesis, we employed a murine hind limb ischemia model to assess how ADAMTS13 deficiency affects both collateral vessel growth and capillary formation. We evaluated perfusion recovery, vascular cell proliferation, immune cell infiltration, and thrombotic activity in ADAMTS13^−/−^ and wild-type control mice to determine whether ADAMTS13 is essential in vascular adaptation following arterial occlusion.

## 2. Results

To investigate the role of ADAMTS13 in arteriogenesis and angiogenesis, we employed a murine hindlimb ischemia model in which unilateral femoral artery ligation (FAL) induces the growth of collateral arteries in the adductor muscle of the affected limb [43]. This ligated limb is referred to as the “occluded” side (occ). The contralateral, non-ligated limb served as an internal control to account for surgical manipulation and potential effects of suture placement and is referred to as the “sham” side. FAL reduces perfusion in the lower limb, resulting in ischemia and stimulating angiogenesis in the gastrocnemius muscle. All experiments were conducted in parallel in wild-type (control) and ADAMTS13-deficient (ADAMTS13^−/−^) mice for comparative analysis.

### 2.1. ADAMTS13 Deficiency Does Not Alter Perfusion Recovery

**Figure 1:**
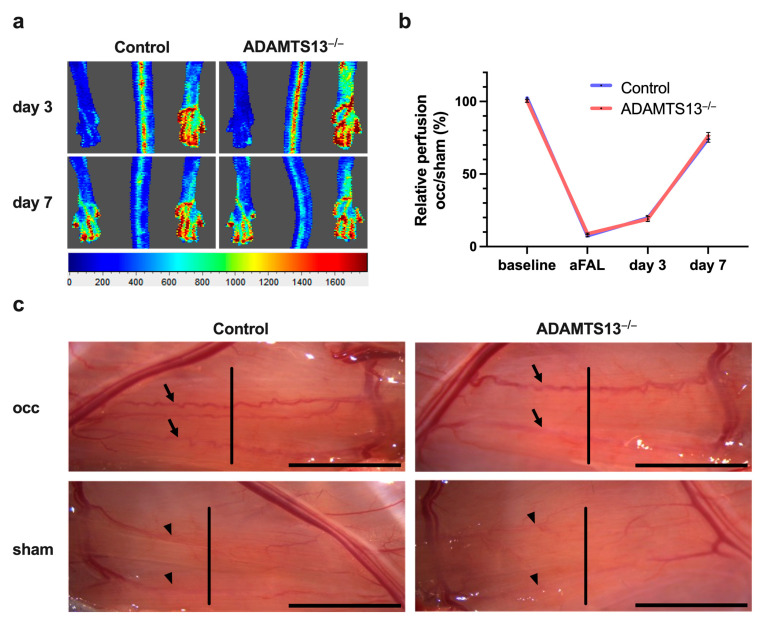
ADAMTS13 deficiency does not impact perfusion recovery after ligation of the femoral artery (FAL). (**a**) Representative flux images of laser Doppler measurements of control (left images) and ADAMTS13^−/−^ mice (right images) 3 days and 7 days after FAL. The flux scale bar is displayed below (red: high flow, blue: low flow). (**b**) Line graph showing the relative perfusion (occ/sham) of control and ADAMTS13^−/−^ mice. The perfusion recovery was calculated before the surgical procedure (baseline), directly after the surgical procedure (aFAL), on day 3, and on day 7 after FAL. No significant differences were observed at any timepoint. Data shown are means ± standard error of the mean (SEM); *n* = 5 mice per group. Control was compared to ADAMTS13^−/−^ by two-way repeated measures ANOVA with Bonferroni’s multiple comparisons test. (**c**) Representative images of superficial collateral arteries taken 7 days after FAL (occ, on the top) or sham operation (on the bottom). The arrows highlight the prominently developed collaterals with typical corkscrew formation in the ligated hindlimb (occ). The arrowheads mark the preexisting, thin, and linear collateral vessels in the non-ligated limb (sham), both of which connect the femoral and profunda femoris arteries. Collateral vessels were cut perpendicular to their course at the mid-portion (indicated by the vertical line in the image) for subsequent staining. Scale bar: 5 mm.

Laser Doppler perfusion measurements revealed that ADAMTS13^−/−^ mice exhibited no significant difference in perfusion recovery compared to wild-type controls at day 3 or 7 after induction of arteriogenesis by femoral artery ligation (Figure 1a,b). Macroscopically, superficial collateral vessels in the adductor muscle appeared similar to those of control mice. In both groups, a prominent corkscrew-shaped collateral artery consistently formed upon FAL between the profunda femoris artery and the superficial femoral artery, assuming normal vascular remodeling and collateralization. The preexisting collaterals, however, appeared thin and linear, in stark contrast to the newly formed bypasses (Figure 1c).

### 2.2. Collateral Vascular Remodeling Is Preserved in ADAMTS13-Deficient Mice

**Figure 2:**
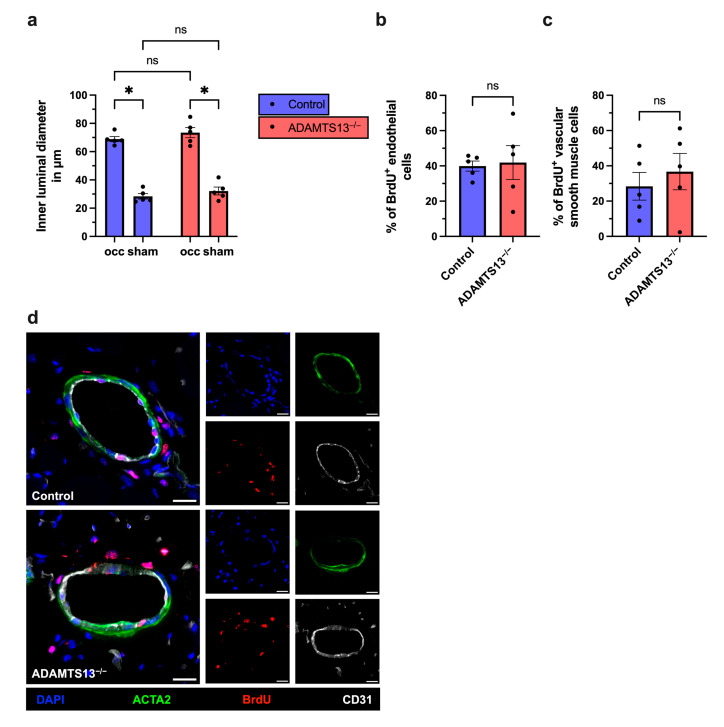
ADAMTS13 deficiency does not affect collateral artery cell proliferation 7 days after femoral artery ligation. (**a**) Inner luminal diameter of growing and resting collateral arteries in all four groups (wild-type controls and ADAMTS13^−/−^ mice, each with ligated (occ) and sham-operated sides). Statistical analysis was performed using a two-way repeated measures ANOVA with Bonferroni’s multiple comparisons test. Percentage of (**b**) BrdU^+^ endothelial cells (CD31^+^/DAPI^+^) relative to the total number of endothelial cells and (**c**) BrdU^+^ smooth muscle cells (ACTA2^+^/DAPI^+^) relative to the total number of smooth muscle cells in collateral arteries of the occluded side in control and ADAMTS13^−/−^ mice. Groups were compared using an unpaired Student’s *t*-test. Data shown are means ± SEM; *n* = 5 mice per group; * *p* < 0.05, and ns *p* ≥ 0.05. (**d**) Representative immunofluorescence images of growing collateral arteries illustrate merged (left) and single-channel (right) for control (upper panels) and ADAMTS13^−/−^ (lower panels) mice. BrdU (red) marked proliferating cells, ACTA2 (green) labeled smooth muscle cells, CD31 (white) visualized endothelial cells, and DAPI (blue) stained nuclei. In the merged images, proliferating cells appear pink due to overlay of BrdU (red) and DAPI (blue). Scale bar: 20 µm.

To determine whether preserved perfusion recovery in ADAMTS13^−/−^ mice reflects effective collateral remodeling, we performed immunofluorescence analysis on adductor muscle tissue 7 days after FAL. Endothelial cells were labeled with an anti-CD31 antibody to assess luminal diameter. In both ADAMTS13^−/−^ and wild-type (control) mice, FAL induced a significant increase in collateral artery diameter compared to the sham-operated contralateral side, indicating successful arteriogenic remodeling. However, no significant difference in the luminal diameter was observed between the two groups (Figure 2a,c).

To distinguish between vasodilation and structural remodeling, we evaluated vascular cell proliferation by bromodeoxyuridine (BrdU) incorporation. Co-staining with ACTA2, a marker for smooth muscle cells, allowed the identification of proliferating vascular cells within the collateral vessel wall. Both ADAMTS13^−/−^ and wild-type control mice showed a comparable number of BrdU^+^ cells in collaterals on the ligated side, while no BrdU^+^ cells were detected on the sham-operated side (Figure 2b–d and Supplementary Material, Figure S1). When analyzed separately, endothelial cells and smooth muscle cells revealed similar proliferation rates in ADAMTS13^−/−^ and wild-type control mice (Figure 2b,c). These results show that ADAMTS13 deficiency does not impair positive vascular remodeling or vascular cell proliferation during arteriogenesis.

### 2.3. Macrophage Recruitment and Polarization Are Unaffected by ADAMTS13 Deficiency During Arteriogenesis

Given the central role of macrophages in arteriogenesis, we analyzed macrophage recruitment and polarization by immunofluorescence staining on adductor muscles 7 days after induction of arteriogenesis by femoral artery ligation. This timepoint represents the active remodeling phase during collateral growth when macrophage recruitment and polarization are most pronounced [16]. CD68 was used as a general macrophage marker, while mannose receptor C-type 1 (MRC1) served to distinguish between M1-like (MRC1^−^) and M2-like (MRC1^+^) macrophages.

**Figure 3:**
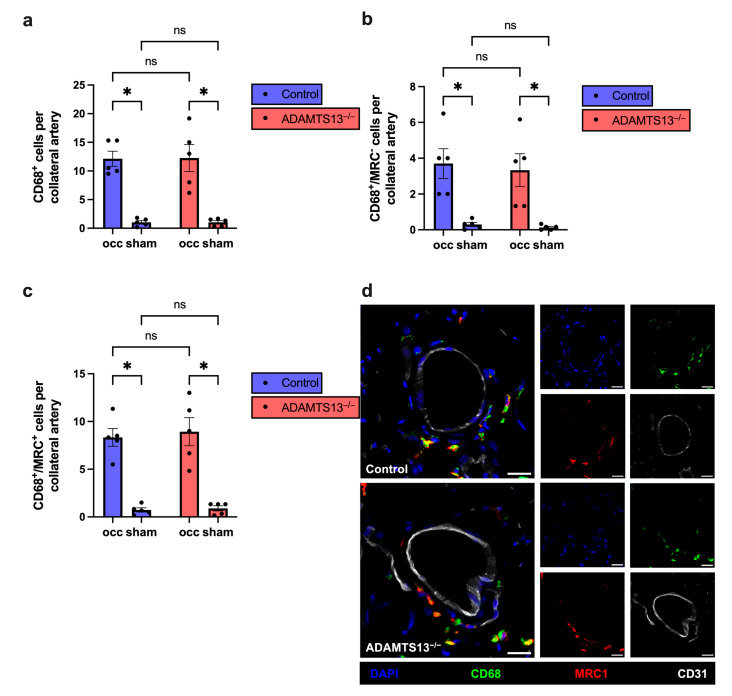
ADAMTS13 deficiency does not impair macrophage recruitment or polarization in arteriogenesis. (**a**) The total number of perivascular macrophages (CD68^+^ cells), (**b**) the number of M1-like macrophages (CD68^+^/MRC1^−^ cells), and (**c**) the number of M2-like macrophages (CD68^+^/MRC1^+^ cells) per growing (occ) and resting (sham) collateral artery of control (blue bars) and ADAMTS13^−/−^ (red bars) mice 7 days after FAL. Data are presented as means ± SEM; *n* = 5 mice per group; * *p* < 0.05, and ns *p* ≥ 0.05. Statistical comparisons were performed using two-way repeated measures ANOVA with Bonferroni’s multiple comparisons test. (**d**) Representative immunofluorescence images of growing collateral arteries are shown as merged images (left) and single stain (right) for control (upper panels) and ADAMTS13^−/−^ (lower panels). CD68 (green) marked macrophages, MRC1 (red) labeled M2-like macrophages, CD31 (white) visualized endothelial cells, and DAPI (blue) stained nuclei. In the merged images, colocalization of CD68 (green) and MRC1 (red) appears as yellow, indicating M2-like macrophages. Scale bar: 20 µm.

Quantitative analysis revealed a significant increase in perivascular macrophage number on the ligated side (occ) compared to the sham-operated side in both control and ADAMTS13^−/−^ mice, consistent with macrophage recruitment during active arteriogenesis. However, no significant differences were observed between genotypes on either the occ or sham sides, indicating that ADAMTS13 deficiency does not affect the extent of macrophage infiltration (Figure 3a,d). Similarly, the numbers of M1-like macrophages (CD68^+^/MRC1^−^) and M2-like macrophages (CD68^+^/MRC1^+^) were comparable between both groups (Figure 3b–d).

Taken together, these findings demonstrate that ADAMTS13 does not modulate macrophage recruitment or polarization during collateral vessel growth.

### 2.4. Endothelial VWF Release Remains Intact in the Absence of ADAMTS13

During arteriogenesis, activation of VEGFR2 rapidly triggers endothelial release of VWF, thereby promoting platelet activation and PNA formation with subsequent mast cell activation and leukocyte recruitment [4,8,9]. To investigate whether VWF is released from endothelial cells of collateral arteries during this early phase of arteriogenesis, we took advantage of the principle that antibodies cannot penetrate intact cell membranes. Accordingly, to detect extracellular VWF, an anti-VWF antibody was administered intravenously prior to tissue collection.

**Figure 4:**
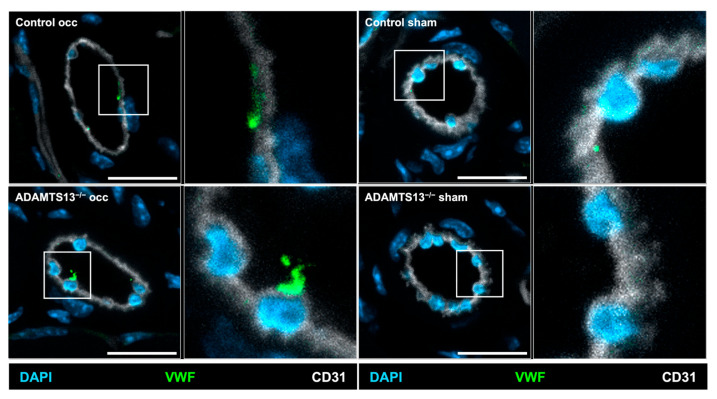
ADAMTS13 deficiency does not inhibit VWF release from endothelial cells of growing collateral arteries. Representative immunohistological stains of collateral arteries showing endothelial cells with VWF on their luminal surface of control (upper panels) and ADAMTS13^−/−^ (lower panels) mice; VWF is only scarcely visible on the sham sides. Anti-VWF (green) served as a marker for VWF, CD31 (white) visualized the endothelial cells, and DAPI (blue) labeled nuclei. The inset on the right displays a magnification of luminal VWF. Scale bar: 20 µm.

Immunohistological analysis performed 2 h after induction of arteriogenesis revealed VWF presence on endothelial cells in growing collateral arteries in both ADAMTS13^−/−^ and control mice, indicating that this process is not impaired by ADAMTS13 deficiency. Figure 4 therefore provides a qualitative assessment of extracellular VWF on collateral endothelium in both groups. In contrast, almost no VWF signal was detected on the sham-operated side in either group, confirming that VWF release is specifically induced by arteriogenic activation (Figure 4).

### 2.5. Platelet-Leukocyte Aggregate Formation and Mast Cell Activation Are Unchanged in ADAMTS13-Deficient Mice

Given that VWF is released at the onset of arteriogenesis, we further assessed whether the early inflammatory and thromboinflammatory events of arteriogenesis are affected by the absence of ADAMTS13. Therefore, we analyzed PNA formation, as a sensitive marker of platelet activation, as well as mast cell recruitment and degranulation 24 h after FAL, a timepoint at which PNA formation and mast cell activation are known to be maximal [8,9].

**Figure 5:**
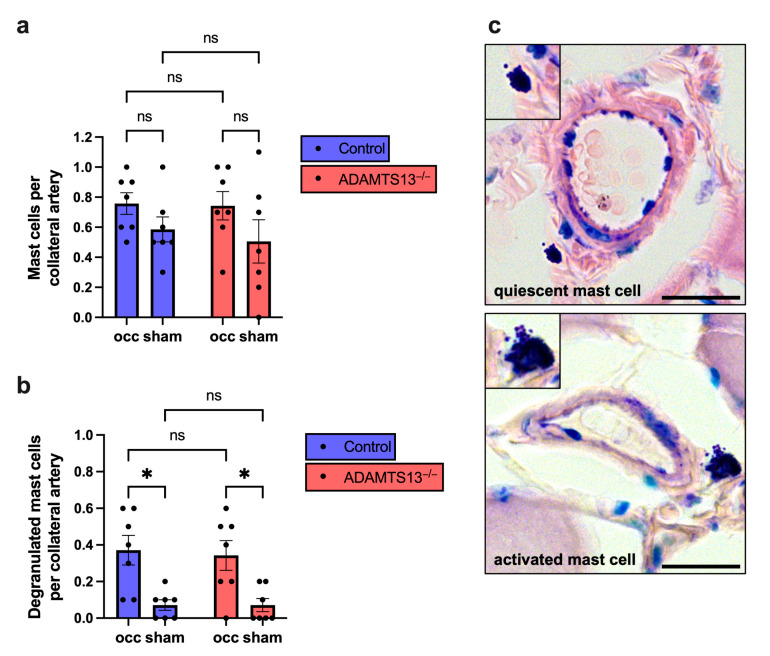
ADAMTS13 has no significant impact on mast cell recruitment and activation. (**a**) Quantification of total perivascular mast cells per growing and resting collateral artery, and (**b**) the number of degranulated mast cells in the same regions in control and ADAMTS13^−/−^ mice 24 h after induction of arteriogenesis by femoral artery ligation, based on Giemsa staining. Data are presented as means ± SEM; *n* = 7 mice per group; * *p* < 0.05, ns *p* ≥ 0.05; analyzed using two-way repeated measures ANOVA with Bonferroni’s multiple comparisons test. (**c**) Representative Giemsa-stained images showing a resting mast cell (top) and a degranulated mast cell (bottom). Insets show magnified views. Scale bar: 20 µm.

Flow cytometry revealed no significant differences in PNA formation or peripheral blood neutrophil counts between ADAMTS13^−/−^ and wild-type mice (Supplementary Materials, Figure S2a–c). Histological analysis of Giemsa-stained adductor muscle sections showed no significant differences in mast cell recruitment between the sham-operated and the ligated sides, nor between ADAMTS13^−/−^ and control mice (Figure 5a). However, mast cell degranulation was significantly increased on the ligated side compared to the sham side in both groups, indicating physiological mast cell activation in response to arteriogenic stimuli. Importantly, no differences were detected between ADAMTS13^−/−^ and wild-type control mice (Figure 5b,c).

These findings suggest that early platelet–neutrophil interactions and mast cell activation proceed normally in the absence of ADAMTS13.

### 2.6. ADAMTS13 Deficiency Increases Platelet Adhesion Without Thrombus Formation

**Figure 6:**
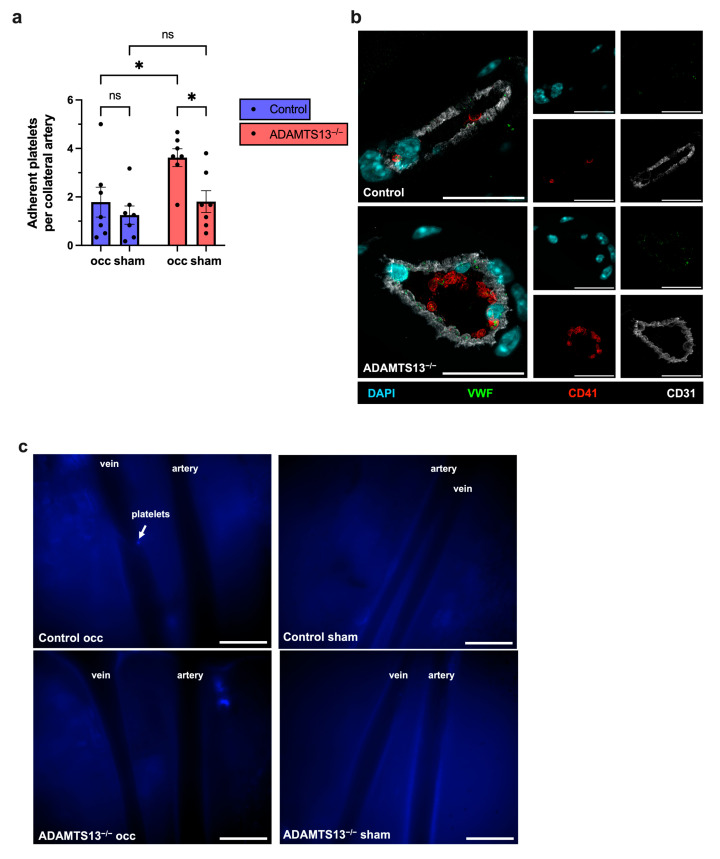
Enhanced platelet adhesion in ADAMTS13^−/−^ mice without thrombus formation. (**a**) Scatter plot of adherent platelets per collateral artery (occ and sham) in control and ADAMTS13^−/−^ mice. Data are presented as means ± SEM; *n* = 7 mice per group; * *p* < 0.05; ns *p* ≥ 0.05; two-way repeated measures ANOVA with Bonferroni’s multiple comparisons correction. (**b**) Representative immunofluorescence images of growing collaterals: merged (left) and single-channel (right) views for control (top) and ADAMTS13^−/−^ (bottom). CD41 (red) marked platelets, VWF (green), CD31 (white) labeled endothelial cells, and DAPI (blue) stained nuclei. Scale bar: 20 µm. (**c**) Representative images from epifluorescence intravital microscopy of collaterals of the occ and the sham side in control (upper panels) and ADAMTS13^−/−^ (lower panels) mice, 24 h after FAL. Veins run alongside the collateral arteries. *n* = 5 mice. Scale bar: 100 µm.

To investigate potential early thrombotic events in ADAMTS13^−/−^ mice, we examined platelet and VWF accumulation in non-perfused tissue 24 h after induction of arteriogenesis by FAL. Immunostaining revealed a significantly increased number of adherent platelets on the vascular endothelium of growing collateral arteries on the ligated side in ADAMTS13^−/−^ mice compared to wild-type controls. Notably, despite this increased adhesion, there was no evidence of complete thrombotic occlusion in either genotype. In sham-operated limbs, platelet adherence remained low and did not differ between ADAMTS13^−/−^ and control mice (Figure 6a,b).

Intravital microscopy using an intravenously administered CD41 antibody to label circulating platelets confirmed the absence of intraluminal thrombi in growing collateral arteries of ADAMTS13^−/−^ and control mice 24 h after FAL. A representative signal of platelet adhesion was observed in a vein on the ligated side of a control animal, providing a visual reference for the typical appearance of adherent platelets, which was not observed in collateral arteries (Figure 6c).

### 2.7. Ischemic Injury Extent Is Not Altered by ADAMTS13 Deficiency

**Figure 7:**
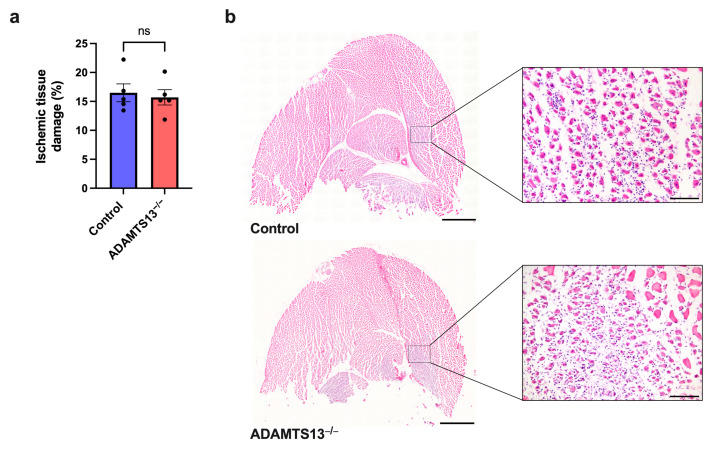
ADAMTS13 deficiency does not alter the extent of ischemic tissue damage. (**a**) Scatter plot showing the percentage of ischemic tissue damage relative to the total gastrocnemius muscle area in control and ADAMTS13^−/−^ mice 7 days after FAL, with one complete cross-sectional area analyzed per mouse. Data are presented as means ± SEM; *n* = 5 mice per group; ns *p* ≥ 0.05; statistical analysis by unpaired Student’s *t*-test. (**b**) Representative images of hematoxylin and eosin (H&E) stained sections of gastrocnemius muscles from control (top) and ADAMTS13^−/−^ (bottom). Scale bars: 1000 µm (overview) and 100 µm (detail).

While our data indicated that arteriogenesis is unaffected by ADAMTS13 deficiency, we next investigated whether the downstream angiogenic response was altered. To assess ischemic tissue damage, hematoxylin and eosin (H&E) staining was performed on gastrocnemius muscle sections collected 7 days after FAL. No differences in the extent of ischemic injury were observed between ADAMTS13^−/−^ and control mice (Figure 7a,b). Sham-operated sides showed no evidence of ischemic damage in either group (representative images are provided in Supplementary Materials, Figure S3).

### 2.8. Capillary Density Is Not Altered by ADAMTS13 Deficiency

To investigate the impact of ADAMTS13 deficiency on ischemia-induced angiogenesis, we performed immunofluorescence staining for CD31, CD45, BrdU, and DAPI on gastrocnemius muscle sections collected 7 days after FAL. Capillaries were specifically quantified by counting CD31^+^/CD45^−^/DAPI^+^ cells, thereby excluding leukocytes that express CD31 but are CD45 positive. Muscle fibers were identified based on their autofluorescence (see Supplementary Materials, Figure S4). The capillary-to-muscle fiber ratio was used as a measure of angiogenic activity.

**Figure 8:**
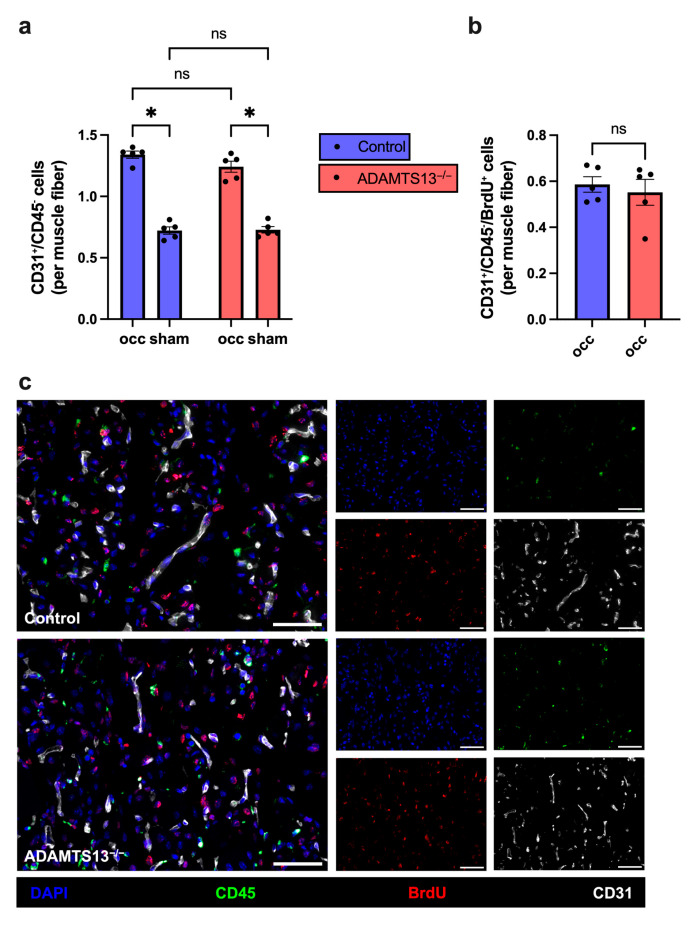
ADAMTS13^−/−^ mice do not show a difference in capillarity 7 days after FAL. (**a**) Number of endothelial cells (CD31^+^/CD45^−^/DAPI^+^) per muscle fiber of ischemic (occ) and non-ischemic (sham) gastrocnemius muscle of control and ADAMTS13^−/−^ mice. Statistical analysis was performed using two-way repeated measures ANOVA with Bonferroni correction for multiple comparisons. (**b**) Proliferating endothelial cells (CD31^+^/CD45^−^/BrdU^+^/DAPI^+^) per muscle fiber in the ischemic region of gastrocnemius muscle in control and ADAMTS13^−/−^ mice. Groups were compared using an unpaired Student’s *t*-test. Data are presented as means ± SEM; *n* = 5 mice per group; * *p* < 0.05; ns *p* ≥ 0.05. (**c**) Representative immunofluorescence staining of ischemic gastrocnemius muscle sections. Merged images (left) and single-channel views (right) are shown for control (upper panels) and ADAMTS13^−/−^ (lower panels) mice. BrdU (red) marked proliferating cells, CD45 (green) labeled leukocytes, CD31 (white) visualized endothelial cells, and DAPI (blue) stained nuclei. In the merged images, proliferating cells appear pink, reflecting BrdU (red) colocalized with DAPI (blue). Scale bar: 50 µm.

Both ADAMTS13^−/−^ and wild-type control mice showed a significantly increased capillary density on the occluded side compared to the sham side, demonstrating effective angiogenic stimulation in response to ischemia. There was, however, no significant difference in capillary-to-muscle fiber ratio between the two genotypes (Figure 8a,c). Similarly, both the number of proliferating endothelial cells (CD31^+^/CD45^−^/BrdU^+^ per muscle fiber) and the proliferation rate (CD31^+^/CD45^−^/BrdU^+^ as a percentage of total CD31^+^/CD45^−^ cells) in the ischemic gastrocnemius muscles were identical in ADAMTS13^−/−^ and wild-type control mice (Figure 8b,c and Supplementary Materials, Figure S5). In contrast, no endothelial cell proliferation was detected in the non-ischemic, sham-operated gastrocnemius muscles of either genotype. Finally, capillary density on sham-operated sides did not differ between the two groups, confirming that ADAMTS13 deficiency does not affect baseline vascularization (Figure 8a).

### 2.9. ADAMTS13 Deficiency Increases Macrophage Recruitment Without Affecting Polarization

Since ADAMTS13 deficiency did not affect macrophage recruitment or polarization during arteriogenesis, we next investigated whether this also held true in the context of angiogenesis. To address this, we again used CD68 as a general marker for macrophages and MRC1 to identify M2-like polarized macrophages.

In ischemic gastrocnemius muscle, total macrophage counts per mm^2^ were significantly higher in ADAMTS13^−/−^ mice than in controls (Supplementary Material, Figure S6a,d). However, the proportions of M1-like and M2-like polarized macrophages did not differ between genotypes (Supplementary Material, Figure S6b–d). Both genotypes showed significantly increased macrophage recruitment in the gastrocnemius muscle of the ligated limbs compared to the non-ligated side. In the sham-operated muscles, no differences were observed between ADAMTS13^−/−^ and control mice regarding either total macrophage number or their polarization (Supplementary Materials, Figure S6a–c).

These findings suggest that, while ADAMTS13 deficiency may enhance macrophage accumulation in ischemic tissue, it does not alter their polarization profile during angiogenesis.

### 2.10. ADAMTS13 Deficiency Does Not Affect Neutrophil Accumulation and NET Formation

Neutrophils and their ability to form neutrophil extracellular traps (NETs) are key components of the early inflammatory response to ischemia. To assess the neutrophil response, gastrocnemius tissue was analyzed 3 days after FAL using immunofluorescence staining for myeloperoxidase (MPO, a neutrophil marker), citrullinated histone H3 (CitH3, a NET marker), and DAPI. Cells positive for both MPO and DAPI were classified as neutrophils, while MPO^+^/CitH3^+^/DAPI^+^ cells were identified as NET-forming neutrophils.

Quantitative analysis showed no significant differences in total neutrophil numbers between ADAMTS13^−/−^ and control mice (Supplementary Figure S7a,c). Similarly, NET density (NETs/mm^2^) was comparable between both genotypes (Supplementary Figure S7b,c). In sham-operated, non-ischemic muscles, only minimal numbers of neutrophils were present, while NETs were absent in both ADAMTS13^−/−^ and control mice, indicating that the presence of neutrophils and NET formation is driven by ischemic conditions (Supplementary Figure S7a).

## 3. Discussion

In this study, we investigated the role of ADAMTS13 in arteriogenesis and angiogenesis using a murine hindlimb ischemia model. While ADAMTS13 is well known for its role in cleaving ULVWF to reduce thrombotic and inflammatory activity, our findings challenge the assumption that it is a critical regulator in collateral artery growth and angiogenesis in peripheral skeletal muscle ischemia [4,44]. Contrary to prior assumptions, our results demonstrate that ADAMTS13 deficiency does not impair the growth of collateral arteries or capillary growth, nor is it associated with thrombus formation in the remodeling vasculature.

Experimental studies have demonstrated that ADAMTS13 deficiency enhances thrombus formation on activated endothelium, an effect reversed by recombinant ADAMTS13, highlighting its protective role [45]. In several models, ADAMTS13 has been shown to exert protective anti-thrombotic and anti-inflammatory effects, for example, in diabetic nephropathy or myocardial pressure overload injury [46,47]. Clinical and experimental studies reveal that low ADAMTS13 plasma levels are associated with an increased risk of myocardial infarction, stroke, or arterial thrombosis in subarachnoid hemorrhage [40,48,49,50,51,52,53]. In a mouse model of ischemic stroke, it was shown that ADAMTS13 can dissolve occlusive thrombus, thus facilitating efficient thrombolysis and vessel recanalization [54].

Based on these findings, we hypothesized that ADAMTS13 deficiency would promote platelet aggregation and thrombus formation in growing collateral vessels, thereby impairing perfusion recovery. However, our data did not support this hypothesis. We found no significant difference in hind limb perfusion recovery between ADAMTS13^−/−^ and wild-type mice. To ensure that perfusion data reflected actual collateral growth, we assessed collateral vessel diameter and endothelial cell proliferation. Both parameters were comparable between the groups, confirming unimpaired arteriogenesis despite ADAMTS13 deficiency.

Although we found increased platelet adhesion in ADAMTS13^−/−^ mice 24 h after ligation, no thrombotic occlusions were detected histologically or by intravital microscopy. Several compensatory mechanisms may explain these findings. First, the elevated platelet adhesion observed histologically may reflect post-mortem effects, as uncleaved ULVWF remains attached to the endothelial surface in the absence of ADAMTS13, facilitating platelet retention during blood flow cessation. Second, local production of nitric oxide (NO), which plays a central role in inhibiting platelet activation under physiological conditions, may compensate for the absence of ADAMTS13, potentially due to shear stress-induced upregulation of NO in collateral vessels [55,56]. Third, it is also plausible that other proteolytic systems, such as leukocyte-derived proteases, compensate for ADAMTS13 loss by degrading ULVWF and limiting thrombus formation [57,58].

To ensure that the early steps of arteriogenesis were not affected by the absence of ADAMTS13, we examined endothelial VWF release at 2 h, as well as PNA formation and mast cell activation 24 h after femoral artery ligation. During the process of arteriogenesis, VEGFR2 activation has been shown to trigger endothelial VWF release, which in turn promotes platelet activation, formation of PNAs, followed by subsequent mast cell activation and leukocyte recruitment [4,8,9]. Our immunostaining demonstrated preserved VWF release in ADAMTS13^−/−^ mice, consistent with previous reports showing that endothelial VWF secretion remains intact in the absence of ADAMTS13 [36,59]. Likewise, PNA formation and mast cell activation were not significantly altered. Although a slight, non-significant increase in PNAs was observed in ADAMTS13^−/−^ mice, possibly reflecting a mildly pro-thrombotic environment, this did not appear to affect downstream inflammatory responses. These findings suggest that the initiation phase of arteriogenesis, including VEGFR2-dependent VWF release, early platelet–leukocyte interactions and mast cell activation, was not significantly disrupted or enhanced in the absence of ADAMTS13.

In several pathological contexts, ADAMTS13 has been shown to modulate inflammation. Mechanistically, ADAMTS13 deficiency has been associated with enhanced leukocyte rolling and adhesion to the endothelium in unstimulated venules, as well as increased neutrophil transmigration into inflamed tissue [36]. These data support the notion that ADAMTS13 regulates not only thromboinflammatory balance but also early leukocyte–endothelial interactions.

Studies in cerebral and myocardial ischemia/reperfusion injury demonstrated larger infarct sizes and increased infiltration of neutrophils and pro-inflammatory cytokines in ADAMTS13-deficient mice, effects that were attenuated by recombinant ADAMTS13 administration [38,39,40]. Comparable observations have been made in models of acute kidney injury, diabetic retinopathy, rheumatoid arthritis, or sickle cell disease, where loss of ADAMTS13 increased inflammation, and recombinant ADAMTS13 exerted anti-inflammatory effects [42,60,61,62]. In particular, studies in ADAMTS13-deficient ApoE^−/−^ mice demonstrated accelerated atherosclerotic lesion development accompanied by increased macrophage infiltration into plaques, highlighting the relevance of ADAMTS13 in modulating vascular inflammation [37,63]. Based on the more pronounced inflammatory responses observed in various disease models of ADAMTS13 deficiency, we hypothesized that its absence might also affect arteriogenesis, a process that critically depends on the balance between pro- and anti-inflammatory signaling. However, our data showed that ADAMTS13 deficiency does not alter the number of perivascular macrophages, nor does it affect the polarization toward pro-inflammatory (M1-like) or pro-regenerative (M2-like) macrophage phenotypes. These findings suggest that the absence of ADAMTS13 does not significantly contribute to a pro-inflammatory shift during sterile inflammation associated with arteriogenesis.

In our model, FAL induces arteriogenesis in the upper hindlimb, and as a consequence of reduced perfusion, it causes ischemic tissue damage in the lower limb, particularly in the gastrocnemius muscle [43,64]. Given that ADAMTS13 deficiency has no impact on collateral artery growth, we investigated whether it might alter the downstream ischemic injury by analyzing tissue damage in the gastrocnemius muscle. Quantification of ischemic damage revealed no significant differences between ADAMTS13^−/−^ and control mice. It was demonstrated that enhanced arteriogenesis correlates with reduced ischemic injury in the lower limb [9]. Accordingly, this finding of no significant difference in overall ischemic injury is consistent with our observation of preserved arteriogenesis in the upstream femoral collateral network, which ensures timely restoration of perfusion and thereby limits ischemic injury in both groups.

Previous in vitro studies have suggested a role for ADAMTS13 in modulating angiogenesis through effects on the VEGF/VEGFR2 signaling pathway. Recombinant ADAMTS13 has been shown to promote angiogenesis in human umbilical vein endothelial cells (HUVECs) in a dose-dependent manner by enhancing tube formation, proliferation, and migration in vitro. Interestingly, when ADAMTS13 was administered together with VEGF, the VEGF-induced angiogenic response showed attenuation rather than enhancement. This inhibitory effect was attributed to a direct interaction between the thrombospondin type 1 (TSP1) domain of ADAMTS13 with VEGF, suggesting a dual modulatory role of the metalloprotease [65]. In a subsequent study, ADAMTS13 was shown to rapidly upregulate VEGF mRNA and protein expression and to enhance VEGFR2 phosphorylation via its C-terminal TSP1 repeats. Blocking VEGF signaling abrogated these effects, confirming the VEGF dependence of ADAMTS13-induced endothelial activation [66]. Supporting these in vitro observations, knockdown of endogenous ADAMTS13 in HUVECs resulted in significantly impaired proliferation, migration, and tube formation, likely due to reduced VEGF levels, further indicating a pro-angiogenic role of ADAMTS13 independent of its enzymatic activity against VWF [67]. These findings suggest that ADAMTS13 plays a context-dependent role in angiogenesis, with both pro-angiogenic and VEGF-inhibitory effects depending on the signaling environment. In contrast to these in vitro data, our results demonstrate that the absence of ADAMTS13 does not impair angiogenesis in vivo. Capillary density and endothelial cell proliferation were not significantly reduced in ADAMTS13^−/−^ mice compared to wild-type controls.

Based on the reported anti-inflammatory role of ADAMTS13, we hypothesized that loss of ADAMTS13 may lead to excessive inflammation during ischemia-induced angiogenesis with increased leukocyte infiltration in the ischemic muscle. Indeed, we observed a significant increase in total macrophage infiltration within the ischemic gastrocnemius muscle of ADAMTS13^−/−^ mice. However, no difference in macrophage polarization was detected, with comparable proportions of pro-inflammatory M1-like and pro-regenerative M2-like macrophage subsets between the genotypes. Furthermore, neutrophil infiltration into the ischemic muscle following femoral artery ligation was not elevated in ADAMTS13-deficient animals. This contrasts with findings from reperfusion-based models such as cerebral ischemia, where ADAMTS13 deficiency led to enhanced neutrophil-driven injury, suggesting that its role in ischemic inflammation may depend on the specific pathophysiological context.

Collectively, our data indicate that ADAMTS13 deficiency does not significantly alter ischemia-related angiogenesis in skeletal muscle. In this arteriogenesis-driven model, where perfusion is progressively restored through collateral artery growth, both VEGFR2-dependent endothelial proliferation and inflammation-mediated processes of angiogenesis appear to proceed normally, without evidence of impairment.

Overall, these findings regarding arteriogenesis and angiogenesis suggest that the modulatory effects of ADAMTS13 may be more relevant in other models, such as acute reperfusion injury, rather than in settings characterized by gradual vascular adaptation and recovery.

The clinical implications of these findings are particularly relevant for peripheral artery disease (PAD). Studies have reported reduced ADAMTS13 activity or elevated VWF/ADAMTS13 ratios in young patients with atherosclerosis, including those with PAD. Notably, the combination of low ADAMTS13 levels and elevated VWF concentrations has been associated with the highest risk for arterial events [68]. Our results suggest that, despite reduced ADAMTS13 activity, the ability to form collateral vessels and capillaries may remain intact, at least in the absence of acute thromboinflammatory events. This could imply that endogenous vascular adaptation, such as the growth of natural bypasses, may not be critically impaired in PAD patients with low ADAMTS13 levels.

Limitations of this study include the possibility that ADAMTS13-deficient mice may compensate differently than humans, which makes it challenging to directly extrapolate our findings to clinical practice. Moreover, our model simulates arterial occlusion in non-atherosclerotic vessels, unlike the atherosclerotic context of peripheral artery disease in humans. This difference could alter the role of ADAMTS13 and affect the applicability of our results.

In conclusion, our findings refine the current understanding of ADAMTS13’s role in ischemic revascularization. While ADAMTS13 is critical in limiting thrombus formation in acute thromboinflammatory settings such as stroke or TTP, it appears to be dispensable for collateral artery and capillary growth in models of gradual perfusion recovery driven by arteriogenesis.

## 4. Materials and Methods

### 4.1. Animals

All experiments involving animals were conducted in accordance with the German Animal Welfare Act and the NIH guidelines on animal care. Ethical approval was obtained from the Government of Upper Bavaria (Regierung von Oberbayern, Germany; approval code: ROB-55.2–2532.Vet_02–22–99; approval date: 29 March 2023), and experiments were performed in compliance with the institutional guidelines of the Walter Brendel Centre of Experimental Medicine. The mice were maintained under standardized environmental and housing conditions, including regulated temperature and humidity, with a 12 h light/dark cycle, and were provided with a standard laboratory diet. Environmental enrichment consisted of coarse bedding, play tunnels, gnawing sticks, cellulose tissues, and cellulose swabs.

To examine the influence of ADAMTS13 on arteriogenesis and angiogenesis, 8–12-week-old ADAMTS13-deficient male mice (ADAMTS13^−/−^ on a C57BL/6J background; B6.129-Adamts13^tm1Dgi^/J, The Jackson Laboratory, JAX strain #007235), provided by S. Schneider from the University Medical Center Hamburg-Eppendorf and bred in-house, were compared to age-matched wild-type C57BL/6J male mice (referred to as control), either obtained from Charles River Laboratory (Sulzfeld, Germany) or bred at the institute. Animals were included in the experiment no earlier than four days after arrival to allow sufficient time for acclimatization. Only animals in full health were enrolled. Endpoints were defined a priori based on a standardized score sheet, which was used for daily monitoring. Assessed parameters included body weight, fecal output, behavior, general condition and grooming, signs of pain, wound healing, and complications of the vascular occlusion. No animals had to be excluded based on these criteria.

### 4.2. Femoral Artery Ligation, Laser-Doppler Perfusion Measurements, and Tissue Sampling

To induce arteriogenesis in the adductor muscle and angiogenesis in the gastrocnemius muscle, the murine hind limb ischemia model was employed as previously described [43,64]. Mice were anesthetized using a combination of fentanyl (0.05 mg/kg, Dechra Veterinary Products Deutschland GmbH, Aulendorf, Germany), midazolam (5.0 mg/kg, Ratiopharm GmbH, Ulm, Germany), and medetomidine (0.5 mg/kg, Pfister Pharma, Berlin, Germany) (MMF). A minimally invasive surgical procedure was performed to ligate the right femoral artery just distal to the origin of the epigastric artery. To control for surgical manipulation and suture placement, a sham operation was conducted on the contralateral femoral artery, where the artery was exposed and a suture placed beneath it without ligation (see Supplemental Figure S8). Postoperative analgesia consisted of buprenorphine (0.1 mg/kg, Dechra Veterinary Products Deutschland GmbH), administered subcutaneously at a volume of 0.1 mL 10 min before awakening from anesthesia and every 8 h on postoperative days 1, 2, and 3.

Hind limb perfusion was assessed using laser Doppler imaging (LDI) (Moor LDI 5061 and Moor Software Version 3.01, Moor Instruments, Axminster, Devon, UK) at baseline (preoperatively), immediately after ligation (aFAL), and on postoperative days 3 and 7. To prevent hypothermia and minimize temperature-related variability, measurements were conducted in a temperature-controlled chamber at 36–38 °C. Imaging began 10 min after the mice were placed in the chamber. The region of interest (ROI) was defined in the software and set to 0.42 cm^2^ for both hind limbs across all experimental groups (see Supplemental Figure S9). A mean flux value was calculated, and perfusion was quantified as the ratio of flux in the ligated limb (occ) to the sham-operated limb (sham).

Tissue was collected at 2 h, 24 h, 3 or 7 days following femoral artery ligation. For 24 h, 3- and 7-day timepoints, mice were re-anesthetized with MMF. Blood was collected by cardiac puncture using a heparinized syringe, yielding 500–1000 μL of blood for flow cytometry. Following blood collection, animals were euthanized by cervical dislocation under deep anesthesia. For mast cell staining, PNA analysis, and platelet adhesion, *n* = 7 mice per group were used; for all other histological staining, *n* = 5 mice per group were analyzed.

For 7-day tissue collection, an aortic catheter was inserted, and the lower body was perfused with 20 mL of adenosine buffer (1% adenosine (Sigma-Aldrich, St. Louis, MO, USA), 5% bovine serum albumin (BSA, Sigma-Aldrich), diluted in phosphate-buffered saline (PBS)), followed by 20 mL of 3% paraformaldehyde (PFA, Merck, Darmstadt, Germany, diluted in PBS). The adductor and gastrocnemius muscles were dissected and incubated for 1 h in 15% sucrose solution, followed by 12 h in 30% sucrose.

For 2 h and 24 h timepoints, adductor and gastrocnemius muscles were collected without prior aortic perfusion. Dissected muscles were fixed in 3% PFA for 1 h, followed by sequential incubation in 15% and 30% sucrose as described above.

All muscles were embedded in Tissue-Tek compound (Sakura Finetek Germany GmbH, Staufen, Germany) and stored at –80 °C. Cryosections (8 μm thick) were prepared from the adductor and gastrocnemius muscles using a cryostat, with collateral vessels in the adductor muscle sectioned perpendicularly at their mid-portion (see Figure 1c and Supplemental Figure S8).

### 4.3. Histology and Immunohistology

In order to investigate arteriogenesis, two superficial collateral arteries (see Figure 1c) within the adductor muscle were analyzed on both the ligated (occ) and sham-operated side of each mouse by histological and immunofluorescence methods. Immunofluorescence staining was conducted according to established standard protocols, ensuring antibody validation, proper fixation, antibody incubation and detection steps [69,70]. For each side, the two collateral arteries were examined in three tissue sections, and the mean of all six measurements per side was calculated. For mast cell staining, five tissue sections per side were analyzed, each containing two collateral arteries. To assess angiogenesis, five images from the ischemic region of the gastrocnemius muscle were evaluated per mouse, while non-ischemic regions from the contralateral leg served as sham controls. Each image encompassed a defined area of 1.5 mm^2^, and the mean value across these images was calculated for each animal. Tissue samples of the gastrocnemius muscle collected seven days after FAL were used to stain the ischemic area for endothelial cells and macrophages, while samples obtained 3 days post-ligation were used to detect neutrophils and NETs.

BrdU Staining: This staining protocol was used to detect proliferating endothelial (BrdU^+^/CD31^+^/DAPI^+^) and smooth muscle cells (BrdU^+^/ACTA2^+^/DAPI^+^) in the collateral vessels. Beginning immediately after femoral artery ligation, mice received daily intraperitoneal injections of bromodeoxyuridine (BrdU; 1.25 mg/day i.p. in 100 μL PBS, Sigma-Aldrich).

Cryosections were incubated for 30 min at 37 °C in 1 M HCl for DNA denaturation, followed by membrane permeabilization with 0.2% Triton™ X-100 (AppliChem GmbH, Darmstadt, Germany) for 10 min. Blocking was performed with 10% donkey serum (ab7475, Abcam, Cambridge, UK), and sections were incubated overnight at 4 °C with the anti-BrdU primary antibody (ab6326, Abcam; 1:100). The next day, donkey anti-rat Alexa Fluor^®^ 555 secondary antibody (A48270, Thermo Fisher Scientific, Waltham, MA, USA; 1:100 in phosphate-buffered saline containing 0.1% Tween-20 (PBS-T)) was applied for 1 h at room temperature (RT). Following a second blocking step with 4% BSA for 30 min, sections were incubated with anti-ACTA2-Alexa Fluor^®^ 488 (F3777, Sigma-Aldrich; 1:400), anti-CD31-Alexa Fluor^®^ 647 (102516, BioLegend, San Diego, CA, USA; 1:100), and DAPI (62248, Thermo Fisher Scientific; 1:1000).

BrdU staining of gastrocnemius muscles was similar, with the modification that FITC-labeled anti-CD45 (11-0451-85, Invitrogen, Waltham, MA, USA; 1:100) was used instead of anti-ACTA2 to distinguish endothelial cells (CD31^+^/CD45^−^/DAPI^+^) from CD31^+^ leukocytes.

Macrophage Staining: This protocol was used to visualize macrophages and assess their polarization, distinguishing pro-inflammatory M1-like macrophages (CD68^+^/DAPI^+^) from pro-regenerative M2-like macrophages (CD68^+^/MRC1^+^/DAPI^+^). Sections were fixed in 4% PFA for 10 min and blocked with 10% donkey serum (ab7475, Abcam) for 1 h at RT. They were then incubated overnight at 4 °C with the anti-MRC1 primary antibody (ab64693, Abcam; 1:200). The next day, donkey anti-rabbit Alexa Fluor^®^ 546 (A10040, Thermo Fisher; 1:200) was applied for 1 h at RT. After a second 30 min blocking step with 4% BSA, sections were stained with anti-CD31-Alexa Fluor^®^ 647 (102516, BioLegend; 1:100), anti-CD68-Alexa Fluor^®^ 488 (ab201844, Abcam; 1:200), and DAPI.

Macrophage staining of gastrocnemius muscles was conducted in the same way, omitting anti-CD31.

Neutrophil and NET Staining: To visualize neutrophils (MPO^+^/DAPI^+^) and NETs (MPO^+^/CitH3^+^/DAPI^+^), gastrocnemius muscle tissue was fixed in 4% PFA for 10 min, permeabilized with 0.5% Triton X-100 (AppliChem GmbH) (In PBS-T) for 2 min at RT, and blocked for 1 h with 10% donkey serum. Sections were incubated overnight at 4 °C with anti-citrullinated histone H3 (Cit-H3, ab5103, Abcam; 1:100) and anti-myeloperoxidase (MPO, AF3667, R&D Systems, Minneapolis, MN, USA; 1:100) in donkey serum. The next day, sections were incubated with the secondary antibodies donkey anti-goat Alexa Fluor^®^ 546 (A11056, Thermo Fisher Scientific; 1:100) and donkey anti-rabbit Alexa Fluor^®^ 488-conjugated secondary antibody (A32790, Thermo Fisher Scientific; 1:200) in PBS-T for 1 h at RT, along with DAPI for nuclear counterstaining.

Platelet Staining: To visualize luminal platelets (CD41^+^) and VWF, cryosections were fixed in 4% PFA for 5 min, permeabilized with 0.2% Triton X-100 (AppliChem GmbH) for 10 min, and blocked with 10% donkey serum (ab7475, Abcam) for 1 h at RT. Sections were then incubated overnight at 4 °C with an anti-VWF antibody (A0082, Dako Denmark/Agilent Technologies, Santa Clara, CA, USA; 1:100). The following day, sections were incubated for 1 h at RT with a donkey anti-rabbit Alexa Fluor^®^ 488-conjugated secondary antibody (A32790, Thermo Fisher Scientific; 1:200), followed by a second 30 min blocking step with 4% BSA. Subsequently, PE anti-mouse CD41 (133906, BioLegend; 1:100), anti-CD31-Alexa Fluor^®^ 647 (102516, BioLegend; 1:100), and DAPI were applied.

VWF Staining: Mice received 800 µg/kg of anti-VWF antibody (A0082, Dako/Agilent Technologies, dissolved in PBS) intravenously (total volume 100 µL) 30 min before tissue collection. Two hours after FAL, tissues were harvested and cryopreserved. Cryosections were stained with donkey anti-rabbit Alexa Fluor^®^ 488 (A32790, Thermo Fisher Scientific; 1:200) for 1 h at RT, followed by blocking with 4% BSA and staining with anti-CD31-Alexa Fluor^®^ 647 (102516, BioLegend; 1:100) and DAPI.

Mast Cell Staining: Mast cells were visualized using standard Giemsa staining protocol as previously described [71]. In brief, tissue sections were fixed in 4% PFA for 5 min, then rehydrated through a descending ethanol series: 5 min each in 100%, 96%, and 70% ethanol. Sections were subsequently incubated for 1 h at 65 °C in Giemsa staining solution (8 mL Giemsa’s azure-eosin-methylene blue solution in 192 mL distilled water). After staining, slides were rinsed in distilled water and briefly differentiated in 0.5% acetic acid, followed by 2 s in 96% and then 100% ethanol. Finally, slides were cleared in xylene and mounted.

Ischemic Area: Tissue damage in the gastrocnemius muscle was evaluated using hematoxylin and eosin (H&E) staining, according to previously described protocols [72]. In brief, tissue sections were fixed in 4% PFA for 5 min, rinsed briefly in distilled water, and stained with Mayer’s hematoxylin for 6 min. After washing under running tap water for 5 min, sections were sequentially incubated in 70% ethanol for 1 min, 0.1% eosin for 5 min, 96% ethanol for 1 min, 99% ethanol for 1 min, and finally in xylene for 2.5 min.

Image acquisition: For image acquisition, a Leica epifluorescence microscope (DM6 B, Leica Microsystems, Wetzlar, Germany) with a 40x objective lens for imaging collateral vessels and a 20× objective for imaging ischemic areas in gastrocnemius muscle was used. ImageJ software, version 2.16.0/1.54g (National Institutes of Health, Bethesda, MD, USA; open-source) was used for cell quantification. The platelet staining was imaged with an LSM 880 (Carl Zeiss AG, Oberkochen, Germany) equipped with an Airyscan detector and a 40× oil immersion objective. Deconvolution post-processing was performed with ZEN 2.3 Black software (Carl Zeiss AG), resulting in improved image quality. Imaging of VWF staining was performed using a Zeiss LSM 980 (Carl Zeiss AG) confocal microscope (Carl Zeiss AG). Post-processing consisted solely of orthogonal projection using ZEN 3.6 Blue software (Carl Zeiss AG).

### 4.4. Intravital Microscopy

The adductor muscle was surgically prepared to expose the collateral branches of the profunda femoris artery, as previously described [73]. Following anesthesia with MMF, mice were positioned on a heating pad with all four limbs immobilized. Modeling clay was placed under the adductor muscle to achieve a flat orientation of the vessels. The skin around the scar from the initial surgery was incised and sutured to form a pocket. Perimuscular fat and superficial muscle covering the collaterals were carefully removed. Throughout the procedure and imaging process, the tissue was kept moist using pre-warmed saline or ultrasound gel. The depth of anesthesia was continuously monitored and maintained with repeated MMF administration. For platelet labeling, 10 μg of Brilliant Violet 421^TM^-conjugated anti-CD41 antibody (133912, BioLegend), diluted in PBS (total volume 100 μL), was injected via the tail vein prior to imaging. Directly after imaging, mice were euthanized by cervical dislocation under deep anesthesia.

Epifluorescence imaging was carried out using a Zeiss LSM 980 (Carl Zeiss AG, Oberkochen, Germany) microscope equipped with an Axiocam 506 mono camera and a Colibri 7 LED excitation system. Fluorescence excitation was achieved using a 385 nm LED, and emission was detected using the 425–430 nm band-pass of the filter. Imaging was conducted with a 20×/1.0 DIC water immersion objective. ZEN 3.6, Blue software (Carl Zeiss AG) was used for image acquisition. Collateral vessels, including both artery and vein, were imaged at the branching point from the profunda femoris artery. Platelet adherence was manually quantified with ImageJ. For imaging, *n* = 5 mice per group were used.

### 4.5. Flow Cytometry

Blood was collected via cardiac puncture using a heparinized syringe (Ratiopharm GmbH). 100 μL of whole blood was lysed in 2 mL BD FACS^TM^ Lysing Solution (349202, BD Biosciences, Franklin Lakes, NJ, USA; diluted 1:9 in distilled water), followed by centrifugation at 400× *g* for 5 min at RT. The supernatant was discarded, and cells were resuspended in an antibody mix of 100 μL 1% BSA in PBS containing the antibodies PE anti-mouse CD11b (101208, BioLegend; 1:300), Brilliant Violet^TM^ 421 anti-mouse CD115 (135513, BioLegend; 1:300), FITC anti-mouse CD41 (133903, BioLegend; 1:400), APC anti-mouse Ly-6G/Ly-6C (108412, BioLegend; 1:800), and eFluor^TM^ 780 viability dye (65-0865-14, Invitrogen; 1:1000).

Samples were incubated for 20 min at 4 °C, centrifuged again (400× *g*, 5 min, RT), and resuspended in 300 μL 1% BSA in PBS. Flow cytometry was performed using the BD LSRFortessa (BD Biosciences, Lakes, NJ, USA). The PE-Texas Red detector (615/20 nm) was left unoccupied to measure background fluorescence and assist in population gating. Data were analyzed with FlowJo^TM^ V10 (BD Biosciences).

### 4.6. Genotyping of ADAMTS13^−/−^ Mice

Genomic DNA was extracted from tissue samples (ear biopsies) using the KAPA Express Extract kit (KE7102, Kapa Biosystems, Wilmington, MA, USA) according to the manufacturer’s instructions. Genotyping was performed using two separate PCR reactions to distinguish ADAMTS13 wild-type and knockout alleles. PCR set A contained Primer 2 (ADAMTS13 oIMR8009, wild common reverse, 5′-GAG TTG CTA GGT TAT CAG GAA G-3′) and Primer 3 (ADAMTS13 oIMR8010, mutant forward, 5′-TGG TTC TAA GTA CTG TGG TTT CC-3′), producing a 290 bp amplicon corresponding to the knockout allele. PCR set B contained Primer 2 and Primer 1 (ADAMTS13 oIMR8008, wild-type forward, 5′-AGC CCC AAC TCT TGT CTT TTA AT-3′), producing a 361 bp amplicon corresponding to the wild-type allele. Each PCR reaction (25 µL) contained 12.5 µL KAPA2G Fast HotStart Genotyping Mix (KK5621, Kapa Biosystems), 10.5 µL nuclease-free water, 0.5 µL of each primer, and 1 µL genomic DNA. PCR cycling conditions were 95 °C for 3 min; 35 cycles of 95 °C for 15 s, 58 °C for 15 s, 72 °C for 30 s; and a final extension at 72 °C for 30 s. PCR amplicons were separated on a 1.5% agarose gel with a 100 bp DNA ladder. Genotypes were assigned as follows: a band in PCR set A only indicated ADAMTS13^−/−^, a band in PCR set B only indicated wild-type (see Supplemental Figure S10).

### 4.7. Statistical Analysis

All statistical analyses were conducted using GraphPad Prism 10 (GraphPad Software, La Jolla, CA, USA). Group comparisons and statistical tests are specified in the respective figure legends. Data are presented as mean ± standard error of the mean (SEM), and *p*-values < 0.05 were considered statistically significant. In cases with no variability (e.g., all sham values = 0), *t*-tests were used instead of two-way ANOVA.

## Data Availability

The data presented in this study are available on request from the first author.

## Supplementary Materials

The following supporting information can be downloaded at: https://www.mdpi.com/article/10.3390/ijms26189137/s1.

## Abbreviations

The following abbreviations are used in this manuscript:

ADAMTS13	A Disintegrin and Metalloprotease with Thrombospondin type 1 repeats, member 13
BrdU	Bromodeoxyuridine
CitH3	Citrullinated Histone H3
FAL	Femoral Artery Ligation
H&E	Hematoxylin and Eosin
HUVEC	Human Umbilical Vein Endothelial Cells
LDI	Laser Doppler imaging
MMF	Midazolam, Medetomidine and Fentanyl
MRC1	Mannose Receptor C-type 1
MPO	Myeloperoxidase
NET	Neutrophil Extracellular Trap
NO	Nitric Oxide
ns	not significant
occ	occluded
PAD	Peripheral Artery Disease
PBS	Phosphate-Buffered Saline
PFA	Paraformaldehyde
PNA	Platelet-Neutrophil Aggregates
ROI	Region Of Interest
RT	Room Temperature
SEM	Standard Error of the Mean
TNFα	Tumor Necrosis Factor-Alpha
TSP1	Thrombospondin Type 1
TTP	Thrombotic Thrombocytopenic Purpura
ULVWF	Ultra Large Von Willebrand Factor
VEGF	Vascular Endothelial Growth Factor
VWF	Von Willebrand Factor
