# Supplementary material for: An Expendable Player in Positive Vascular Remodeling? ADAMTS13 Deficiency Does Not Affect Arteriogenesis or Angiogenesis
Source: Int J Mol Sci. 2025 Sep 19;26(18):9137. doi: 10.3390/ijms26189137 (PMC12471135; doi:10.3390/ijms26189137)
Supplement: Supplementary file 1 [file ijms-26-09137-s001.zip › ijms-3827799-supplementary.pdf]

International Journal of
Molecular Sciences

MDPI

Article

An Expendable Player in Positive Vascular Remodeling? ADAMTS13 Deficiency Does Not Affect Arteriogenesis or Angiogenesis

Carolin Baur [1,2], Amanda Geml [1,2], Kira-Sofie Wimmer [1,2], Franziska Heim [1,2], Anja Holschbach [1,2], Katharina Elbs [1,2], Michael R. Rohrmoser [1,2], Dominic van den Heuvel [1,3], Alexander T. Bauer [4], Stefan W. Schneider [4], Daphne Merkus [1,5], Elisabeth Deindl [1,2,*]

1 Institute of Surgical Research, Walter Brendel Centre of Experimental Medicine, University Hospital, Ludwig-Maximilians-Universität München, 81377 Munich, Germany; carolin.baur@med.uni-muenchen.de (C.B.); amanda.geml@med.uni-muenchen.de (A.G.); kira.wimmer@med.uni-muenchen.de (K.-S.W.); franziska.heim@med.uni-muenchen.de (F.H.); katharina.elbs@med.uni-muenchen.de (K.E.); michael.rohrmoser@med.uni-muenchen.de (M.R.R.); dominic.van@med.uni-muenchen.de (D.v.d.H.); daphne.merkus@med.uni-muenchen.de (D.M.)
2 Biomedical Center, Institute of Cardiovascular Physiology and Pathophysiology, Faculty of Medicine, Ludwig-Maximilians-Universität München, 82152 Planegg-Martinsried, Germany
3 Department of Medicine I, Ludwig-Maximilians-University School of Medicine, 81377 Munich, Germany
4 Department of Dermatology and Venereology, University Medical Center Hamburg-Eppendorf, 20246 Hamburg, Germany; a.bauer@uke.de (A.T.B.); st.schneider@uke.de (S.W.S.)
5 Division of Experimental Cardiology, Department of Cardiology, Thoraxcenter, Erasmus MC, University Medical Center Rotterdam, 3015 GD Rotterdam, The Netherlands
* Correspondence: elisabeth.deindl@med.uni-muenchen.de; Tel.: +49-(0)-89-2180-76504

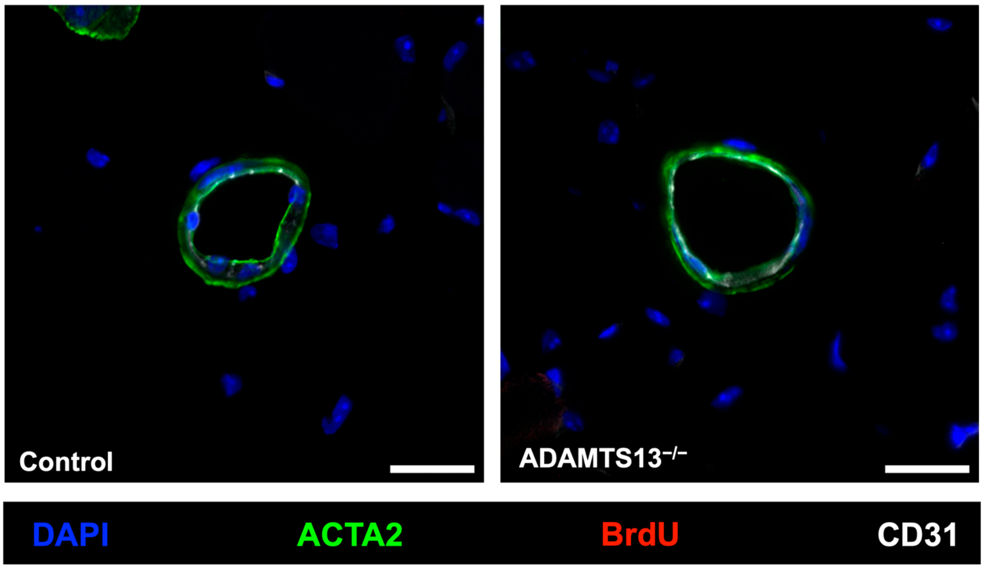

Figure S1. Neither control nor ADAMTS13−/− mice showed BrdU+ proliferating vascular cells in resting collateral arteries 7 days after sham operation. Representative immunofluorescence images of resting collateral arteries from control (left) and ADAMTS13−/− (right) mice. BrdU (red) marked proliferating cells (absent in these images), ACTA2 (green) labeled smooth muscle cells, CD31 (white) visualized endothelial cells, and DAPI (blue) stained nuclei. Scale bar: 20 µm.

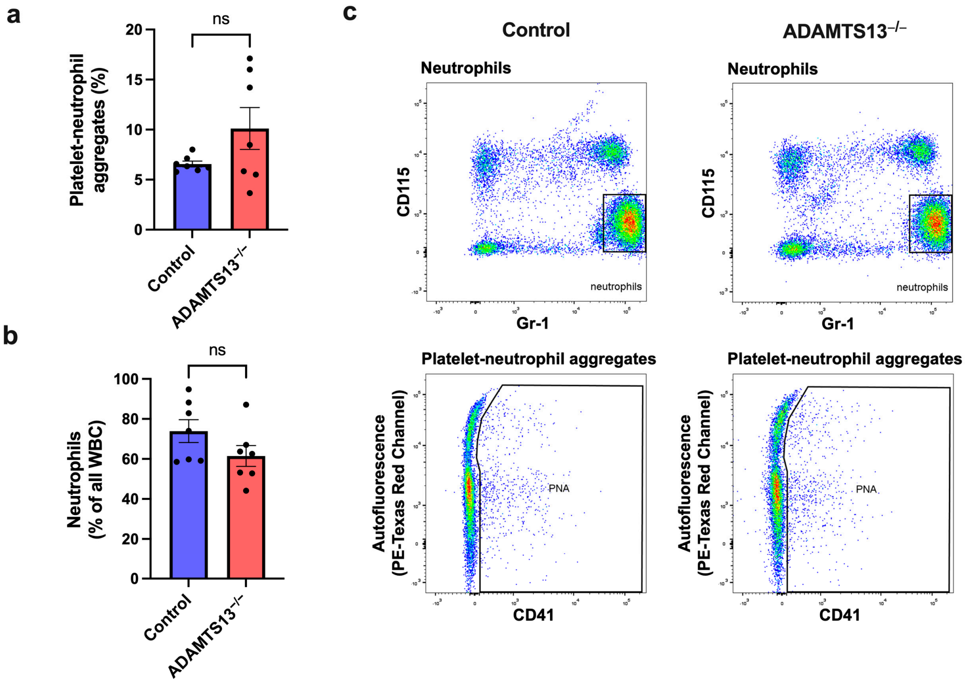

Figure S2. ADAMTS13 has no significant impact on platelet-neutrophil aggregate (PNA) formation 24 hours after induction of arteriogenesis. (**a**) Scatter plot showing the percentage of PNAs relative to the total number of neutrophils and (**b**) the percentage of neutrophils relative to all white blood cells, as assessed by flow cytometry in control and ADAMTS13$^{-/-}$ mice. Data are presented as means ± SEM; $n = 7$ mice per group; not significant (ns): $p \geq 0.05$. Statistical comparison by unpaired Student's t-test. (**c**) Representative flow cytometry plots showing the gating strategy.

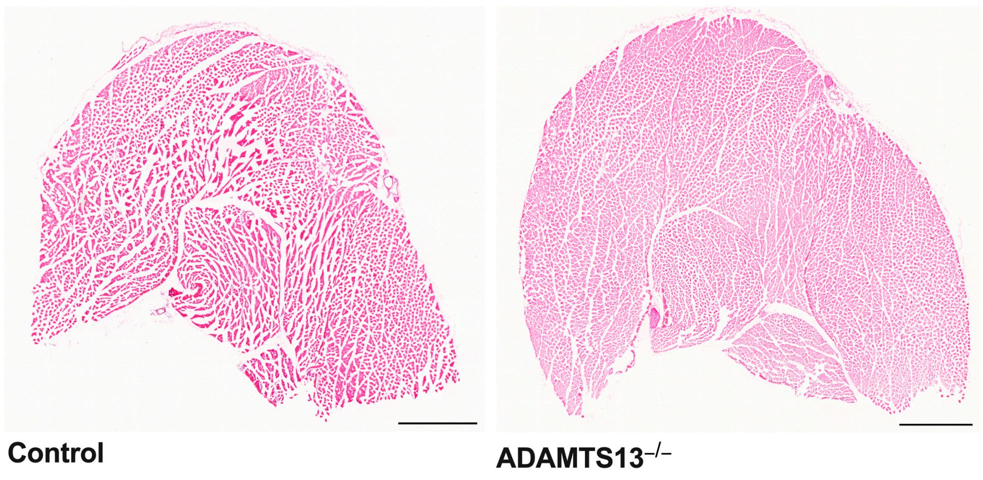

Figure S3. Absence of ischemic injury in sham-operated gastrocnemius muscles. Representative images of H&E-stained sections of gastrocnemius muscles from control (left) and ADAMTS13$^{-/-}$ (right) do not show ischemic tissue damage 7 days after sham operation. One complete cross-sectional area was analyzed per mouse. Scale bar: 1000 μm.

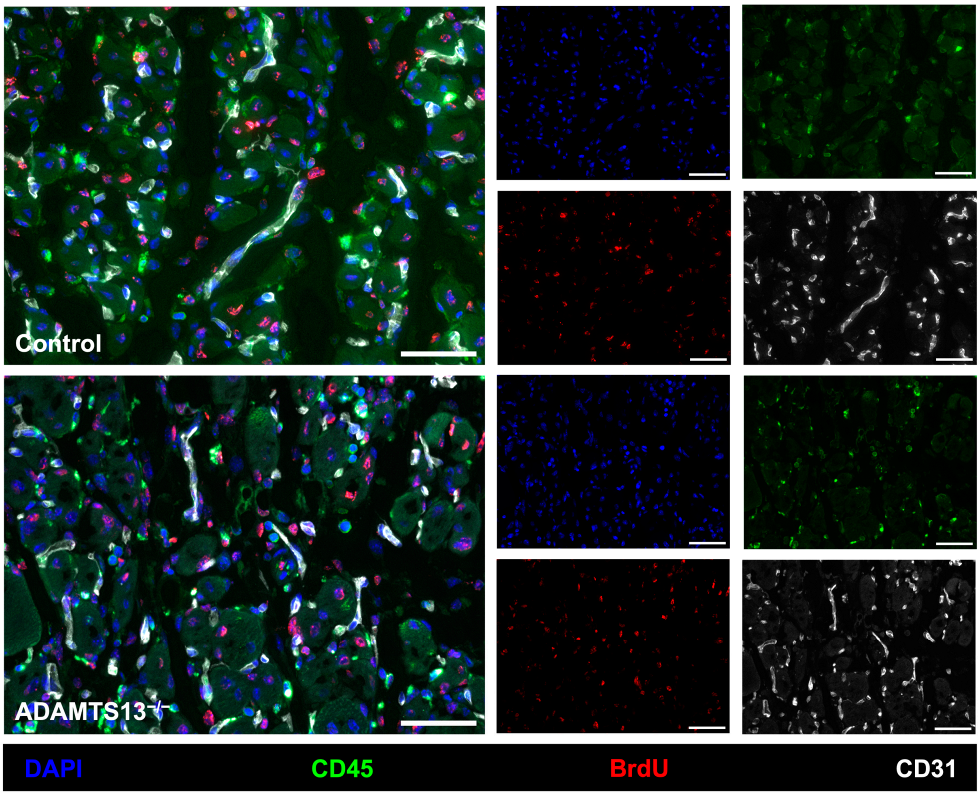

Figure S4. Autofluorescence-based identification of muscle fibers for quantification of capillary density. Representative immunofluorescence staining of ischemic gastrocnemius muscle sections 7 days after FAL. Panels show merged images (left) and single-channel views (right) for control (upper panels) and ADAMTS13$^{-/-}$ (lower panels) mice. CD31 (white) marked endothelial cells, CD45 (green) labeled leukocytes, DAPI (blue) stained nuclei, and muscle fibers are visualized by their intrinsic autofluorescence. The capillary-to-muscle fiber ratio was calculated based on CD31^{+}/CD45^{-}/DAPI^{+} endothelial cells relative to the number of autofluorescent muscle fibers. In the merged images, proliferating cells are visualized in pink, resulting from BrdU (red) colocalization with DAPI (blue). Scale bar: 50 μm.

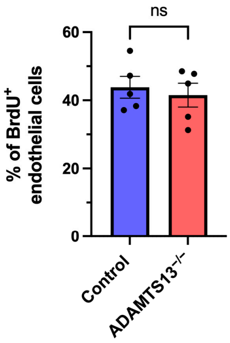

Figure S5. Proliferation rate of endothelial cells during angiogenesis in ischemic gastrocnemius muscle. Shown is the percentage of BrdU^{+} endothelial cells (CD31^{+}/CD45^{-}) relative to the total number of endothelial cells in control and ADAMTS13$^{-/-}$ mice 7 days after FAL. Groups were compared using an unpaired Student's *t*-test. Data are shown as mean ± SEM; $n = 5$ mice per group; ns: $p \geq 0.05$.

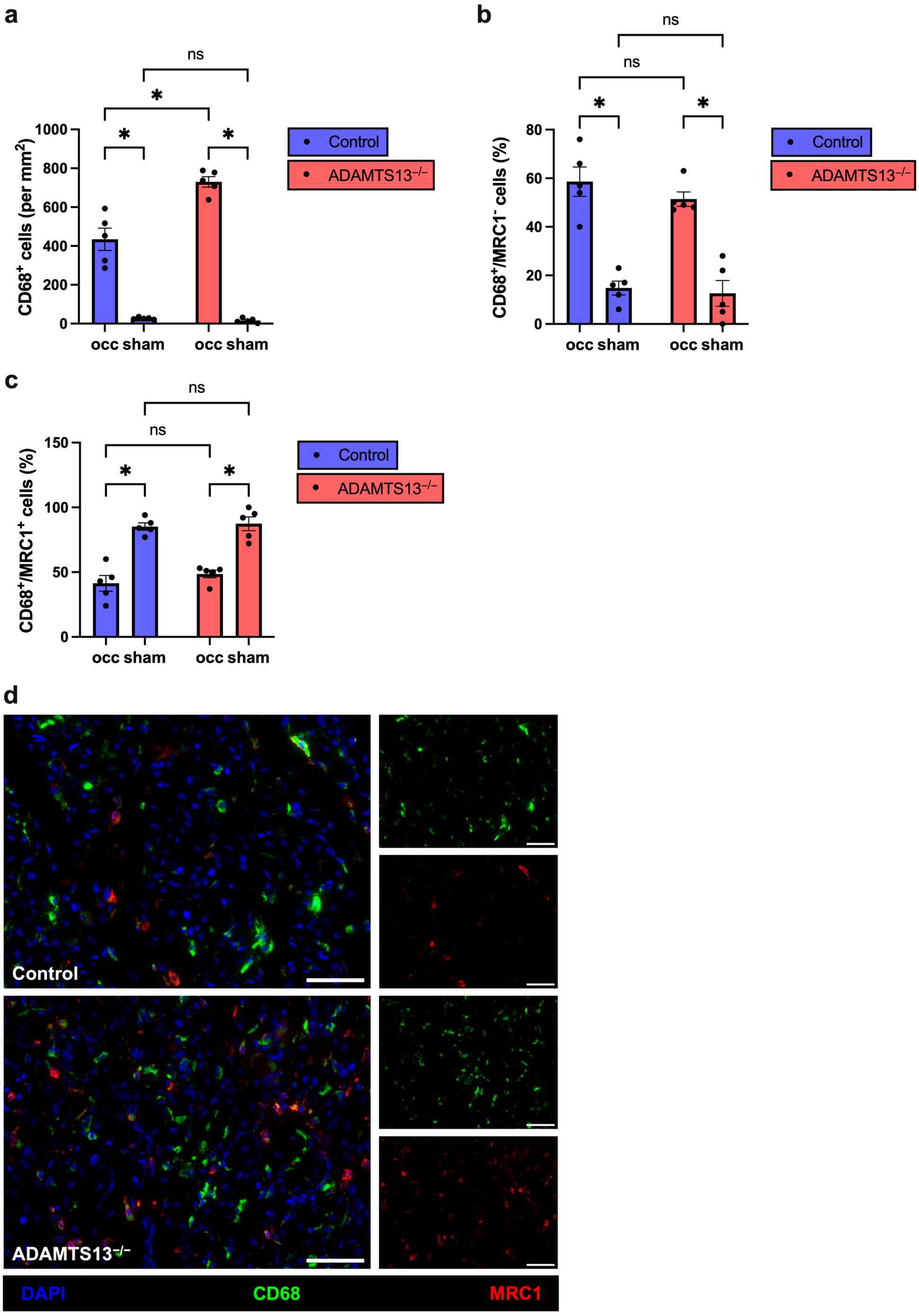

Figure S6. ADAMTS13 deficiency leads to higher recruitment of macrophages but no change in polarization in ischemic tissue. The scatter plots with bars display (**a**) the total number of macrophages ($CD68^+$ cells) per mm^2, (**b**) the percentage of M1-like macrophages ($CD68^+/MRC1^-$ cells), and (**c**) the percentage of M2-like macrophages ($CD68^+/MRC1^+$ cells) in ischemic tissue damage and the sham side of the gastrocnemius muscle in control and $ADAMTS13^{-/-}$ mice 7 days after FAL. Data are presented as means ± SEM; n = 5 mice per group; * $p < 0.05$; ns $p \geq 0.05$; control was compared to $ADAMTS13^{-/-}$ by two-way repeated measures ANOVA with Bonferroni's multiple comparison test. (**d**) Representative immunofluorescence images of analyzed ischemic gastrocnemius muscle tissue. Merged images (left) and single-channel views (right) are shown for control (upper panels) and $ADAMTS13^{-/-}$ (lower panels) mice. CD68 (green) labeled macrophages, MRC1 (red) served as a marker for M2-like macrophages, and DAPI (blue) stained nuclei. Colocalization of CD68 (green) and MRC1 (red) appears as yellow in the merged images, corresponding to M2-like macrophages. Scale bar: 50 μm.

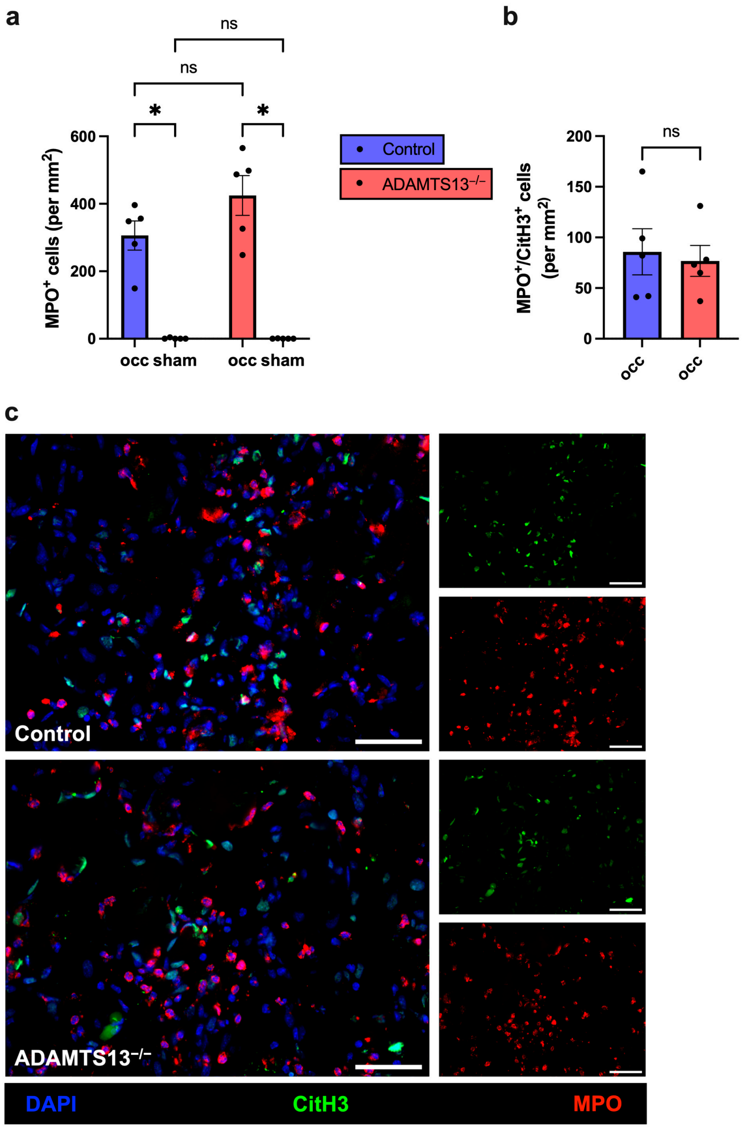

Figure S7. Neutrophil infiltration and NET formation are not altered by ADAMTS13 deficiency. (**a**) Total number of neutrophils ($MPO^+/DAPI^+$ cells) in ischemic (occ) and non-ischemic (sham) gastrocnemius muscle of control and $ADAMTS13^{-/-}$ mice per mm^2. Statistical analysis was performed using two-way repeated measures ANOVA with Bonferroni correction for multiple comparisons. (**b**) Number of NETs ($MPO^+/CitH3^+/DAPI^{+-}$ cells) in the ischemic region of gastrocnemius muscle in control and $ADAMTS13^{-/-}$ mice per mm^2. Groups were compared using an unpaired Student's *t*-test. Data are presented as means ± SEM; $n = 5$ mice per group; * $p < 0.05$; ns $p \geq 0.05$. (**c**) Representative immunofluorescence images of ischemic gastrocnemius muscle tissue. Merged images (left) and single-channel views (right) are shown for control (upper panels) and $ADAMTS13^{-/-}$ (lower panels) mice. MPO (red) served as a marker for neutrophils, CitH3 (green) labeled NETs, and DAPI (blue) stained nuclei. Scale bar 50 µm.

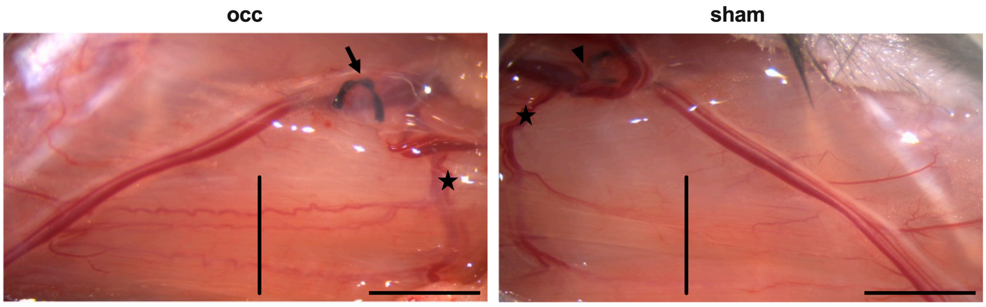

Figure S8. Location of femoral artery ligation. Representative photographs of the collateral arteries 7 days after femoral artery ligation (occ, left) and after sham operation (right). The arrow indicates the ligation site on the femoral artery, placed distal to the origin of the profunda femoris artery (marked with a star). In the sham-operated limb, the arrowhead marks the suture passed beneath the femoral artery without tying a knot at the same location. Collaterals were sectioned perpendicularly at their mid-zone (indicated by the vertical line in the image) for staining. Scale bar: 5 mm.

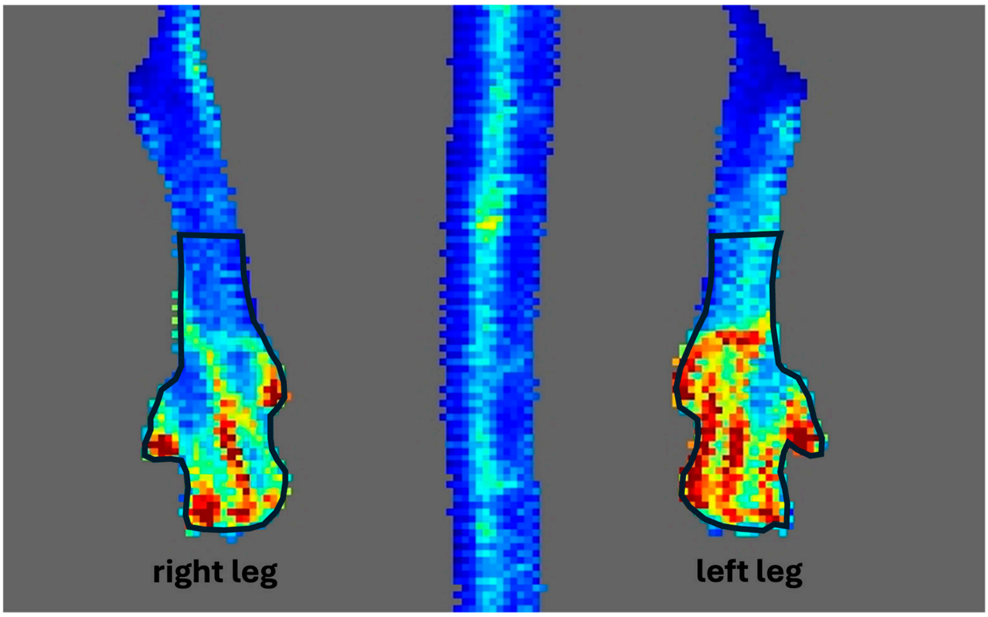

Figure S9. Region of interest (ROI) placement for perfusion analysis in laser Doppler imaging (LDI) measurements. Representative LDI image of hind limbs from a control mouse 7 days after FAL. ROIs were manually defined over the ligated (on the right) and the sham (on the left) leg for quantitative perfusion analysis.

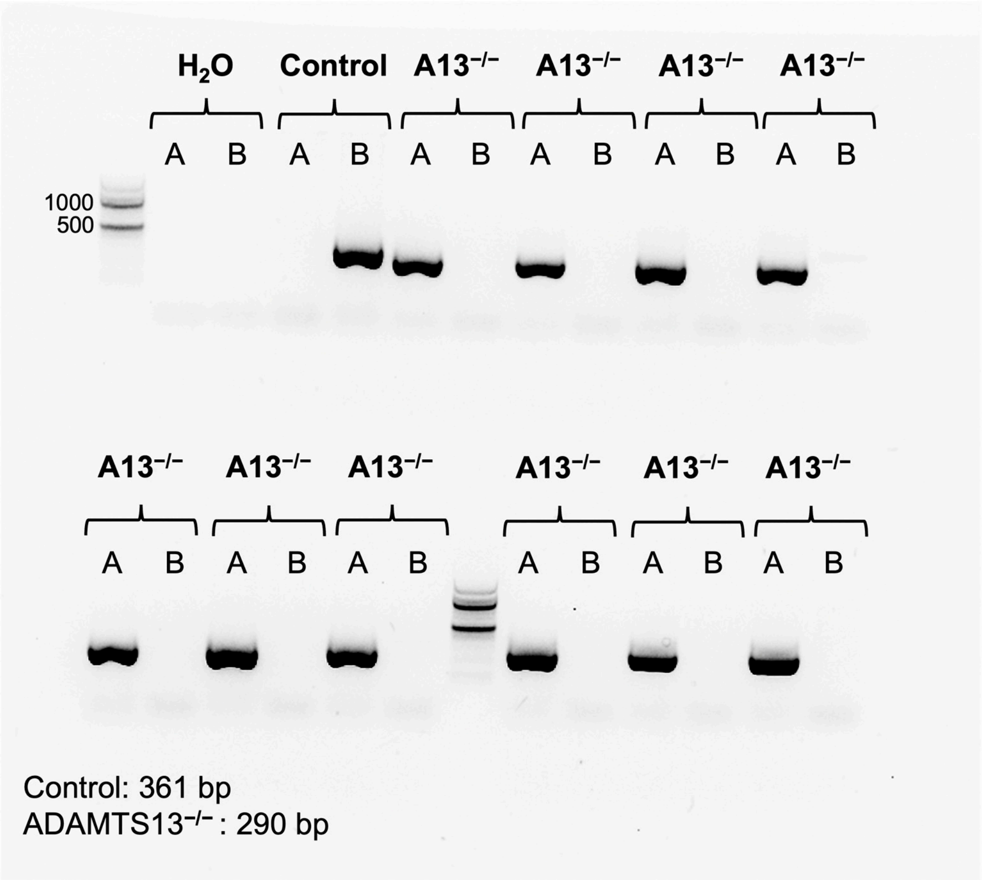

Figure S10. Representative agarose gel showing PCR-based genotyping of ADAMTS13$^{-/-}$ mice. The wild-type (control) allele produces a 361 bp band in PCR set B only, whereas the knockout (A13$^{-/-}$) allele generates a 290 bp band in PCR set A only. A 100 bp ladder was used as size marker.

Disclaimer/Publisher's Note: The statements, opinions and data contained in all publications are solely those of the individual author(s) and contributor(s) and not of MDPI and/or the editor(s). MDPI and/or the editor(s) disclaim responsibility for any injury to people or property resulting from any ideas, methods, instructions or products referred to in the content.
